# Mechanically strengthened graphene-Cu composite with reduced thermal expansion towards interconnect applications

**DOI:** 10.1038/s41378-019-0059-0

**Published:** 2019-05-20

**Authors:** Zhonglie An, Jinhua Li, Akio Kikuchi, Zhuqing Wang, Yonggang Jiang, Takahito Ono

**Affiliations:** 1 Graduate School of Engineering, Tohoku University, Aramaki-Aza-Aoba 6-6-01, Aoba-ku, Sendai 980-8579 Japan; 2 Research Institute for Engineering and Technology, Tohoku Gakuin University, Tagajo, 985-8537 Japan; 3 School of Mechanical Engineering and Automation, Beihang University, Beijing, 100191 PR China; 4 Present Address: Department of Mechanical Systems Engineering, Tokyo University of Agriculture and Technology, Koganei, 184-8588 Japan

**Keywords:** Electrical and electronic engineering, Carbon nanotubes and fullerenes

## Abstract

High-density integration technologies with copper (Cu) through-silicon via (TSV) have emerged as viable alternatives for achieving the requisite integration densities for the portable electronics and micro-electro-mechanical systems (MEMSs) package. However, significant thermo-mechanical stresses can be introduced in integrated structures during the manufacturing process due to mismatches of thermal expansion and the mechanical properties between Cu and silicon (Si). The high-density integration demands an interconnection material with a strong mechanical strength and small thermal expansion mismatch. In this study, a novel electroplating method is developed for the synthesis of a graphene-copper (G-Cu) composite with electrochemically exfoliated graphenes. The fabrication and evaluation of the G-Cu composite microstructures, including the microcantilevers and micromirrors supported by the composite, are reported. We evaluated not only the micromechanical properties of the G-Cu composite based on in-situ mechanical resonant frequency measurements using a laser Doppler vibrometer but also the coefficients of thermal expansion (CTE) of the composite based on curvature radius measurements at a temperature range of 20–200 °C. The Young’s modulus and shear modulus of the composite are approximately 123 and 51 GPa, which are 1.25 times greater and 1.22 times greater, respectively, than those of pure Cu due to the reinforcement of graphene. The G-Cu composite exhibits a 23% lower CTE than Cu without sacrificing electrical conductivity. These results show that the mechanically strengthened G-Cu composite with reduced thermal expansion is an ideal and reliable interconnection material instead of Cu for complex integration structures.

## Circuits: Copper-graphene interconnects

A graphene-copper composite exhibits a coefficient of thermal expansion closer to silicon, as compared to pure copper, suggesting its use for interconnects on circuit boards. Copper is widely used for interconnects in silicon-based electronics due to its high electrical conductivity. However, mismatch in thermal expansion coefficient between copper and silicon generates large internal stresses, potentially causing failure in the long-term. Now, a team led by Takahito Ono from Tohoku University reinforce copper with graphene flakes via an electroplating fabrication approach, achieving higher stiffness and a coefficient of thermal expansion 23% lower than pure copper. These results suggest that this material might enable high-density circuits that can resist internal stresses as a result of thermal expansion.

## Introduction

The booming development of high-density chip-scale packaging (CSP) in portable electronics and system-in-a-package (SiP) of the micro-electro-mechanical systems (MEMSs) industry have faced an increasing demand for advanced interconnection technology. Due to the excellent balance between the cost and the electrical performance, copper (Cu) serves the vast majority of interconnection materials, especially in the traditional interconnection applications of through-silicon-via (TSV), where Cu is integrated in the form of complex microstructures and embedded in typically silicon (Si)-based wafers. However, the dramatic gap in the coefficient of thermal expansion (CTE) between Cu (~17 ppm/K) and Si (~4 ppm/K) can easily introduce thermo-mechanical stresses during the fabrication process of high-density integration structures^1,2^, and the stresses can be converted into normal and shear stresses in response to the temperature variation^3,4^. The residual stresses influence the fatigue life of electronic devices and cause reliability issues. Therefore, the thermal mismatch problem urgently requires an effective solution for decreasing the CTE of Cu. In addition, the practical application also prefers high stretch and shear deformation resistance of interconnection materials for a complex interface motion of TSV structures^5–7^.

Graphene, which is a two-dimensional array of sp^2^-bonded carbon atoms, is known to have a negative CTE in a temperature range of 0–700 K and exhibits ~−8 ppm/K of CTE at room temperature^8,9^. Incorporation of graphene into the Cu matrix can be an effective strategy for decreasing the CTE of the Cu matrix and the thermal stress. Graphene exhibits exceptional mechanical properties, such as the Young’s modulus of ~1 TPa and a tensile strength of ~130 GPa^10–12^. The inclusion of graphene into other materials is expected to enhance the mechanical properties, and numerous graphene-polymer composites have been investigated with epoxy^13–15^, polyurethane^16^, polypropylene^17^ and other polymers. In the area of graphene-metal composites, a few studies have been reported with Cu^18–20^, nickel^21,22^, and magnesium^23^ using reduced graphene oxide or functionalized graphene sheets. Powder metallurgy, spark plasma sintering, electroless plating and electrodeposition were adopted for the synthesis of graphene-metal composite. As a conventionally employed reinforcement in the Cu matrix, graphene-Cu (G-Cu) composites show an improved mechanical strength due to the small grain size of the Cu matrix and uniform dispersion of graphene^18–20^. Proposing G-Cu composite as a potential interconnection material in practical applications, the in-situ analysis of the mechanical properties, such as Young’s modulus and shear modulus, is necessary by setting the G-Cu into actual microstructures. The in-situ resonance technique is an effective method for investigating the dynamic behavior of the composite on a microscale^24,25^. However, related research on G-Cu composite has been rarely reported.

In this study, we developed a new electroplating method of G-Cu composites with electrochemically exfoliated graphenes and fabricated G-Cu composite microstructures combined with Si micromachining to evaluate the mechanical properties of the G-Cu composite. The incorporation of the electrochemically exfoliated graphenes into the Cu matrix has not been reported. The Young’s modulus and shear modulus of the composite are investigated in terms of the resonant frequencies of the cantilever and the micromirror structures, and the rotating angle of the Si micromirror with the composite beams are further evaluated compared with pure Cu beams. In addition, the CTE of G-Cu composite is experimentally investigated at a temperature range of 20–200 °C.

**Fig. 1:**
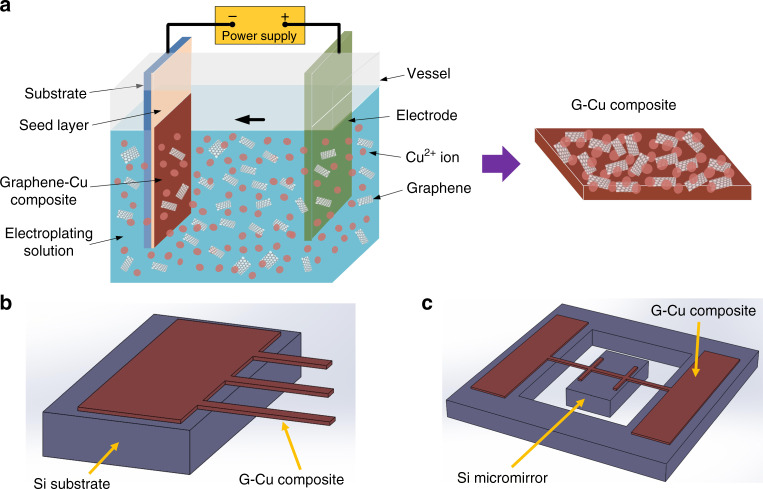
Schematic of (**a**) G-Cu composite electroplating and combination with Si micromachining for (**b**) microcantilever and (**c**) Si micromirror

A schematic of the electroplating setup for synthesizing the composite film is shown in Fig. 1a, and the composite microcantilever array and micromirror are schematically shown in Fig. 1b, c, respectively. The procedure for the exfoliation of graphene, the electroplating of G-Cu composite, and the fabrication of G-Cu composite microstructures and characterizations are described in the Materials and Methods.

## Results and discussion

### Morphology and composition analysis of the G-Cu composite

**Fig. 2:**
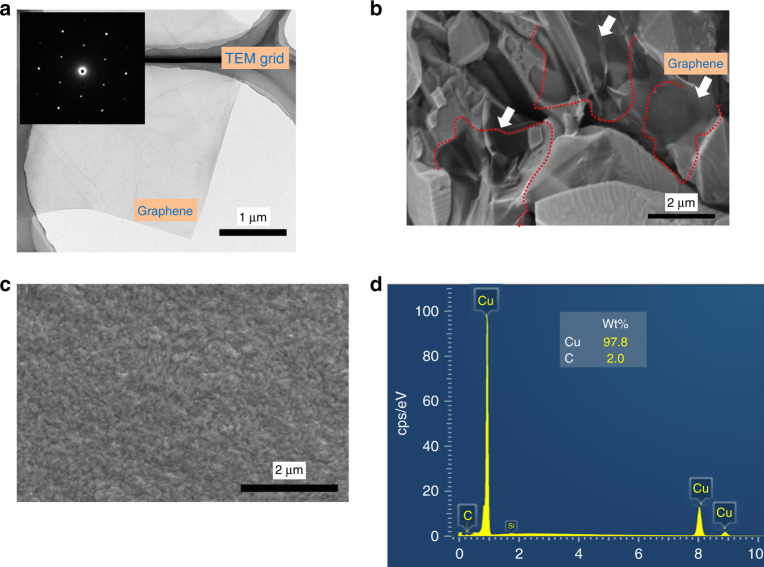
**a** TEM image of the exfoliated graphene on a Cu TEM grid and SAED pattern inset of graphene with high crystallinity, **b** SEM top view image of the G-Cu composite, **c** SEM top view image of Cu thin film, and **d** EDS analysis results of the G-Cu composite

The voltage and electrolyte concentration for electrochemical exfoliation of graphite were chosen to guarantee the quality of the exfoliated graphene^9,26^. A transmission electron microscope (TEM) image of the exfoliated graphene sheet is shown in Fig. 2a. The lateral size was estimated to be 3 μm, as shown in Fig. F1 in supporting information, and the graphene sheet comprised multilayer graphene (mostly 3~4 layers) with subnanometer interlayer spacing in the previous study^26^. The typical 6-fold symmetric selected-area electron diffraction (SAED) pattern in the inset of Fig. 2a exhibits high crystallinity of the exfoliated graphene. The poly(diallyldimethylammonium chloride) (PDDA)-treated graphene with positive charges on the graphene surface effectively avoids graphene aggregation and facilitates the electrodeposition of graphene onto the cathode during the G-Cu composite electroplating. A scanning electron microscope (SEM) image of the electrodeposited G-Cu composite surface and the colorized graphene outline are shown in Fig. 2b. The graphene sheets are embedded into the Cu matrix and the uncovered surface of the graphene is visible, which shows a bumpy morphology with various electrodeposited Cu grains. The electric field concentration near the edge of the graphene flakes causes non-uniform deposition of Cu on the graphene surface during electroplating. Therefore, the Cu matrix is initially deposited at the edge of the graphene and gradually covers the graphene surface, which produces the bumpy morphology. Compared with the pure Cu surface shown in Fig. 2c, the synthesis mechanism of the previously described G-Cu composite is visually verified because obvious crystalline grain is not observed with the pure Cu and the surface roughness of the pure Cu is lower than that of the composite.

The energy dispersive X-ray spectrometry (EDS) analysis with approximately 5 nm of the electron beam diameter and ~3 μm of the depth resolution was performed on the composite surface to determine the weight fraction of the graphene in the G-Cu composite, as shown in Fig. 2d. The average weight fraction of the graphene in the G-Cu composite is calculated more than 15 times in the EDS test.

The weight fractions of the graphene in the composite is 1.9 ± 0.4 wt%. If the densities of Cu and graphene are assumed to be 8.9 g/cm^3^ and 2.3 g/cm^3^,^23^, respectively, the corresponding volume fraction of the graphene in the G-Cu composite is estimated to be 7.0 ± 1.4 vol%. The weight fraction and volume fraction are higher than previously reported values in G-Cu composites by other researchers^18–20^, where the G-Cu composites are synthesized by electrodeposition, spark plasma sintering and electroless plating. In their research, graphene was reduced from graphene oxide by high temperature annealing with an unexpected presence of poorly reduced graphene oxide, oxidized Cu and agglomeration of graphene^18–20^. Conversely, the graphene in this study was directly exfoliated from graphite without annealing and reduction treatments, which may produce higher fractions. The high fraction of graphene is beneficial for efficient mechanical strengthening and CTE reduction, as subsequently discussed.

### In-situ mechanical property measurement of the G-Cu composite

The micromechanical properties of a metal thin film are generally evaluated by performing a microindentation hardness test using a diamond indenter, applying load on the surface of the thin film and plotting the load-depth relationship. The indentation hardness test is beneficial to the evaluation of the static properties of thin films and requires a large number of test times to calculate the average value. Conversely, in this study, the mechanical property of the G-Cu composite thin film is characterized by the in-situ resonant frequency measurement of the cantilever and micromirror. Compared with the indentation hardness test, the dynamic mechanical behaviors of thin films can be investigated by resonant frequency measurement to clarify the practical applications for dynamic devices^24,25^. The fundamental resonant frequencies of flexural and torsional vibration modes of the cantilevers and micromirror are investigated to evaluate the Young’s modulus and shear modulus of the G-Cu composite.

**Fig. 3:**
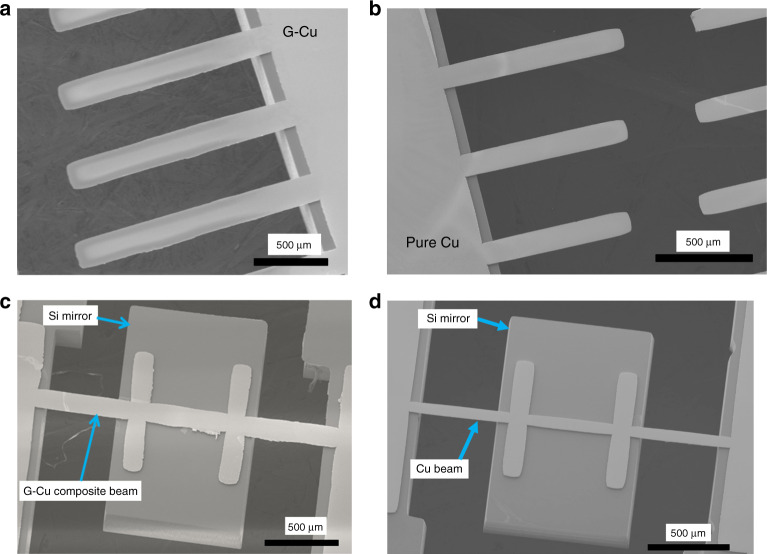
SEM images of the fabricated (**a**) G-Cu composite cantilevers, (**b**) pure Cu cantilevers, (**c**) Si micromirror with G-Cu composite beams and (**d**) Si mirror with pure Cu beams

Typical SEM images of the fabricated G-Cu composite and Cu cantilevers are shown in Fig. 3a, b. As detailed in table S1, both types of the cantilever are fabricated with the same dimension with a length of 400–1700 μm, a width of 50–200 μm and a thickness of 11.5 μm. The surfaces of both cantilevers were grinded by the surface planer to form the same thickness and flat surface. The Cu cantilevers show smoother edges than the G-Cu composite cantilevers in the SEM images.

The SEM images of the fabricated Si micromirrors, which are supported by the G-Cu composite beams and Cu beams, respectively, are shown in Figs. 3c, d. In a similar manner, two kinds of mirror structures were also fabricated with the same dimension listed in table S2. The length, width and thickness of the composite beams in the micromirrors are 500–1000, 50–100, and 11.5 μm, respectively, while, the length, width and thickness of the micromirrors are 800–1000, 1000–1500, and 300 μm, respectively.

**Fig. 4:**
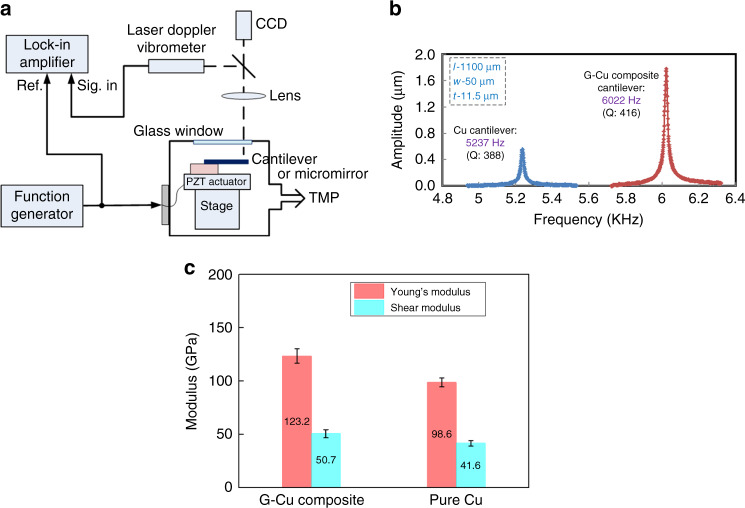
**a** Experimental setup for resonant frequency measurement. **b** Comparison of mechanical frequency responses of the G-Cu composite cantilever with the Cu cantilever. **c** Young’s moduli and shear moduli for the G-Cu composite and pure Cu thin films calculated from the measured resonant frequencies of the microcantilevers and micromirrors

The resonant frequencies at the fundamental resonant mode of the cantilever and the torsional resonant mode of the micromirror were measured using a laser Doppler vibrometer and a lock-in amplifier in the experimental setup shown in Fig. 4a. The microstructures were vibrated using a piezo ceramic actuator with a driving power of 1–2 V at a pressure of 0.5 Pa.

A measurement result of the mechanical frequency responses of the G-Cu composite and pure Cu cantilever is shown in Fig. 4b. The resonant frequency of the composite cantilever is 6022 Hz, which is higher than that of the pure Cu cantilever of 5237 Hz despite the same dimension. The resonant frequency *f*_*c*_ is given by

1 $$f_c = \frac{1}{{2{\mathrm{\pi }}}}\sqrt {\frac{k}{m}} ,$$

where *m* is the effective mass of the cantilever, which is proportional to the actual mass of the cantilever *m*_*c*_ given by *m* = *nm*_*c*_, and *n* is the geometric parameter. The spring constant *k* is given by *k* = *Ewt*^*3*^*/4l*^*3*^, where *E* is the Young’s modulus of the cantilever material; *t, w* and *l* are the thickness, width and length, respectively, of the cantilever. Since the actual mass *m*_*c*_ is given by *m*_*c*_ = *ρtwl*, and the geometric parameter *n* for the rectangular cantilever is 0.24^27^, the relationship between the Young’s modulus *E* and the resonant frequency *f* of the cantilever can be expressed as

2 $$E = \frac{{37.9\rho l^4}}{{t^2}}f_c^2,$$

where *ρ* is the density of the cantilever material. If the densities of Cu and graphene are assumed to be 8.9 and 2.3 g/cm^3^, respectively, the density of the G-Cu composite is calculated to be 8.4 g/cm^3^ from the weight fraction of the graphene, as described in the EDS analysis results.

The Young’s moduli of the G-Cu composite and pure Cu are calculated from the measured resonant frequencies and dimensions of the fabricated several G-Cu composite and pure Cu cantilevers. All data are summarized in table S1. The average Young’s modulus of the composite and the pure Cu are 123 ± 6 and 99 ± 4 GPa, respectively, as shown in Fig. 4c. The Young’s modulus of the composite is approximately 1.25 times greater than that of Cu. This result shows that the embedment of graphene, as observed in Fig. 2b, in the composite increases the Young’s modulus compared with that of pure Cu.

The Young’s modulus enhancement of the G-Cu the composite can be explained by the Voigt-Reuss model for randomly oriented graphene^28^. The Young’s modulus of the G-Cu composite *E*_*f*_ is given by

3 $$E_{nU} = E_cV_c + E_mV_m,$$

4 $$E_{nL} = \frac{{E_cE_m}}{{E_cV_m + E_mV_c}},$$

5 $$E_f = \frac{3}{8}E_{nU} + \frac{5}{8}E_{nL},$$

where *E*_*nU*_ and *E*_*nL*_ are the upper-bound moduli and the lower-bound moduli of the nanocomposite; *E*_*c*_ and *E*_*m*_ are the Young*’*s moduli of graphene and the Young*’*s moduli of Cu; and *V*_*c*_ and *V*_*m*_ are the volume fraction of graphene and the volume fraction of Cu, respectively.

If the Young’s moduli of graphene and Cu are assumed to be 1000 and 99 GPa, the theoretical Young’s modulus of the G-Cu composite with 1.9% weight fractions of graphene in the composites should be 126 GPa based on the Voigt-Reuss model. Compared with the experimentally measured Young’s modulus, the obtained Young’s modulus of the composite is comparable with the theoretical Young’s modulus of the composites. According to the prerequisite of randomly distributed filler in the Voigt-Ruess model, the graphene is uniformly dispersed in the Cu matrix and effectively increases the Young’s modulus, as shown from the dynamic resonance behavior of the composite.

The fundamental torsional resonant frequency *f*_*r*_ of the micromirror is given by

6 $$f_r = \frac{1}{{2\pi }}\sqrt {\frac{{k_t}}{{J_P}}} ,$$

where *J*_*p*_ is the mirror inertia, which depends on the mass and size of the mirror, and *k*_*t*_ is torsional spring constant. The spring constant *k*_*t*_ is given by

7 $$k_t = \frac{{2GJ_t}}{{ls}},$$

where *G* and *l*_*s*_ are the shear modulus of the beam material and the length of the beam, respectively. *J*_*t*_ is the polar moment of area of a non-circular beam. From the two previously described equations, the shear modulus of the beam is given by

8 $$G = \frac{{19.7l_SJ_P}}{{J_t}}f^2,$$

The shear moduli of the G-Cu composite and pure Cu are calculated according to the resonant frequencies and dimensions of the fabricated several micromirrors, as summarized in table S2. The average shear moduli for the composite and the pure Cu are 51 ± 4 and 42 ± 3 GPa, respectively, as shown in Fig. 4c. The shear modulus of the composite is approximately 1.22 times greater than that of the Cu. This result also shows that the embedment of graphene in the composite increases the shear modulus compared with the pure Cu in dynamic conditions.

The randomly embedded graphene in the G-Cu composite improved the mechanical strength for the normal stress and the shear stress. Based on these in-situ characterizations with resonant behaviors of the microstructures, the G-Cu composite exhibits greater mechanical properties than those of the pure Cu due to an excellent combination of graphene and Cu. As observed in Fig. 2b, a large interfacial bonding area of graphene is effective for transferring the stress produced in the Cu matrix to the graphene. Strong interfacial bonding between the graphene and the Cu matrix can be guaranteed by avoiding a severe interfacial reaction^29,30^ and oxygen intermediated chemical bonding on an atomic scale^20^ due to a minute amount of oxygen that originates from the oxidation of graphite during the electrochemical exfoliation^9^.

In addition, the Young’s modulus of the G-Cu composite in this study is similar to previously reported values by other researchers^18–20^. However, the graphene content is higher than that of other studies. The bumpy morphology of the G-Cu composite with various grain sizes of Cu is the main reason since the Young’s modulus of electrodeposited Cu depends on the electrodeposition comdition^18^. The greater mechanical strength of the G-Cu composite would be achieved by improved electrodeposition condition, while the composite possesses a small grain size of Cu and uniform Cu deposition on the graphene surface.

### Electrical properties of the G-Cu nanocomposite

G-Cu composite thin films with two different thickness of 5 and 10 μm on Si substrates were prepared for electrical resistivity measurement by the four-terminal method. The average resistivity of the G-Cu thin film was calculated to be 2.1 ± 0.2 μΩ-cm based on more than 10 times the measurement for each sample. The measured resistivity value is in the range of the values reported for G-Cu composites^18,31^.

The resistivity of the Cu thin film synthesized by electroplating was 1.9 ± 0.1 μΩ-cm, which is slightly lower than that of the G-Cu composites. This result indicates that the addition of graphene in the composite did not significantly affect the resistivity of the composites with a slight degradation in the resistivity of the Cu thin film. Graphene exhibits remarkable electron mobility in the in-plane direction^32^ and electrochemically exfoliated graphene flakes in (NH_4_)_2_SO_4_ aqueous solution showed a conductive sheet resistance in the in-plane direction compared with CVD-grown graphene^9^. However, graphene incorporated in this study is randomly dispersed in the Cu matrix and interfacial bonding between graphene and Cu forms a discontinuous pathway for electron transport in the G-Cu composite although the graphene carries current in other directions. Therefore, the electron transport slowed in the G-Cu composite, which caused a slight degradation in resistivity.

### Thermal properties of the G-Cu nanocomposite

**Fig. 5:**
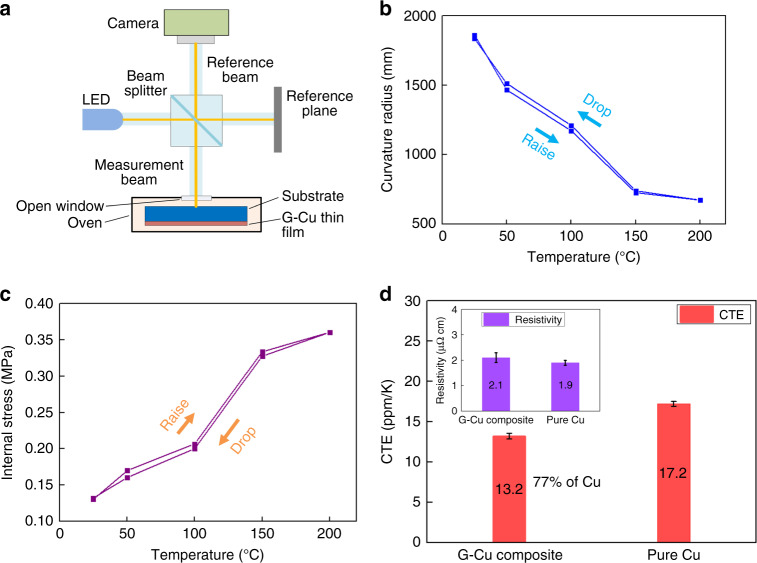
**a** Experimental setup for measurement of curvature radius of the G-Cu composite on substrate according to temperature change. **b** Curvature radius of the G-Cu composite as a function of temperature and **c** thermal stress of the G-Cu composite as a function of temperature and **d** CTEs and electrical resistivities for the G-Cu composite and pure Cu thin films

The CTE of the G-Cu composite thin film was investigated by curvature radius measurement of the composite thin film on Si substrate according to Storney’s formula. The internal stress *σ* of the composite thin film on substrate is given by^33^

9 $$\sigma = \frac{{E_st_s^2}}{{6Rt_f(1 - \nu _s)}},$$

where *E*_*s*_ and *ν*_*s*_ are the Young’s modulus and Poisson’s ratio, respectively, of the substrate; and *t*_*s*_ and *t*_*f*_ are the thickness of the substrate and the composite thin film, respectively. The curvature radius *R* of the composite thin film was measured by the optical interferometer (Polytec Gmbh), while the composite thin film is placed in a constant temperature oven at a temperature range of 20–200 °C, as reported in our previous study and illustrated in Fig. 5a^34^. If the temperature changes, the G-Cu composite thin film would be curved due to the different CTE between the Si substrate and the composite thin film. The CTE difference Δ*α* is given by

10 $$\Delta \alpha = \frac{{\Delta \sigma (1 - \nu _f)}}{{E_f\Delta T}},$$

where Δ*σ* is the change in the thermal stress due to the temperature change Δ*Τ*. *E*_*f*_ is the Young’s modulus for thin film, as cited from the measured Young’s modulus in the mechanical property measurement section. The *E*_*s*_, *ν*_*s*_ and CTE of the Si substrate are referred to the values described in the product information and literature (*E*_*s*_ = 168 GPa and *ν*_*s*_ = 0.34 in the (110) plane, average CTE of Si is 4.0 ppm/K at a range of 20–200 °C)^35–37^. The curvature radius measurement was conducted at 25, 50, 100, 150, and 200 °C, respectively. To analyze the influence of the residual stress relaxation in the composite, the curvature radius was measured in the cases of a temperature increase and temperature decrease.

A 7 μm-thick composite thin film on a 200 μm-thick Si substrate was prepared for the curvature radius measurement, and a Cu thin film on the Si substrate was compared for reference. The G-Cu composite thin film was electrodeposited on a 200 μm-thick Si substrate, and the surface was grinded using a surface planer (DAS8920, DISCO Corporation) with a diamond bit to form a flat surface. The thickness of the thin film was measured by a surface profiler, and 6 times the measurements were conducted. The measured thickness values were 6.9, 7.0, 7.0, 6.8, 7.1, and 7.0 μm, which were averaged to 7.0 + 0.1/−0.2 μm (Figure F4 in supporting information).

The curvature radius of the composite thin film as a function of temperature is shown in Fig. 5b. The curvature radius of the composite films gradually decreased in the period of temperature increase and almost returned to the original state as the temperature gradually decreased to room temperature. The calculated internal stresses of the composite films as a function of temperature are shown in Fig. 5c. If the residual stress relaxation in the composite film changes the curvature, the stress-temperature curve will show hysteresis. As depicted in the graph, the internal stress increased as the temperature increased and almost decreased along the same curve as the temperature decreased. Therefore, the internal stress of the composite film is not affected by the residue stress relaxation in the composite in the measured temperature range.

Based on the experiment, the average CTEs of the G-Cu composite and Cu thin film are measured to 13.2 ± 0.4 and 17.2 ± 0.3 ppm/K, respectively. These results combined with the electrical resistivities described in the electrical property section are plotted in Fig. 5d. The CTE of the G-Cu composite is approximately equal to 77% of the CTE of pure Cu. Thus, the addition of graphene in the G-Cu composite effectively reduces the thermal expansion of Cu, which produces a CTE of the composite that is lower than that of pure Cu. For further estimation, the CTE gap between the interconnection and Si is reduced to (13.2–4.0)/(17.2–4.0) = 70%; 30% of the thermal mismatch stress can be reduced by applying G-Cu composite instead of Cu.

For a comparison between the experimental CTE value of the composite and the theoretical CTE value of the composite, the CTE of the G-Cu composite α_*G-Cu*_ was quantitatively estimated by Turner’s model:^38^

11 $$\alpha _{G - Cu} = \frac{{\alpha _{Cu}K_{Cu}V_{Cu} + \alpha _GK_GV_G}}{{K_{Cu}V_{Cu} + K_GV_G}},$$

where *α*, *K* and *V* are the CTE, bulk modulus and volume fraction, respectively, of the component, respectively. The subscript *G* corresponds to graphene, while *Cu* corresponds to the Cu matrix. The bulk modulus *K* can be calculated using Young*’*s modulus and Poisson*’*s ratio: *K* = *E*/3(1 – 2*ν*). The CTE, Young*’*s modulus and Poisson*’*s ratio of the graphene are referred to the values of single layer graphene from the literature (*α*_G_ = −8 ppm/K, *E*_*G*_ = 1000 GPa, and *ν*_*G*_ = 0.16 for graphene)^10,39–42^. The CTE, Young*’*s modulus and Poisson*’*s ratio of the Cu are cited from the previous experiment and the literature (*α*_*Cu*_ = 17.2 ppm/K, *E*_*Cu*_ = 99 GPa, and *ν*_*Cu*_ = 0.34). According to the previously mentioned parameters, the estimated CTEs for the G-Cu composite is 10.6 ppm/K. The theoretically calculated CTE for the G-Cu composite is approximately 20% lower than the experimentally measured CTE. Because the graphene exfoliated from the graphite and used for the composite electroplating primarily consists of 3~4 multilayer graphene and generally exhibits a higher negative CTE than single layer graphene^41^, the disagreement between the theoretical values and experimental values can be rationalized by the difference in the graphene layer. If single layer graphene is used for the electroplating of G-Cu composite, the CTE of the composite would be further reduced.

According to these characterizations, the electrodeposited G-Cu composite possesses the reduced CTE from the pure Cu without a significant sacrifice in the electrical conductivity. In addition, the in situ mechanical strength of the G-Cu composite film, including the Young’s modulus and shear modulus, is increased from the pure Cu film to render the composite as a mechanically robust and promising interconnection material for CSP, SiP of MEMSs and microelectronics applications. The expected applications can be launched into both static conditions and dynamic conditions of interconnection by solving the safety and reliability issues.

## Conclusions

We presented a synthesis of the G-Cu composite thin film by dispersion electroplating of the composite. The electrochemically exfoliated multilayer graphene was introduced into an electroplating solution, and the weight fraction of graphene in the composite was approximately 1.9 wt%. We also presented a microfabrication of the G-Cu composite, including the microcantilever and micromirror. Based on the in-situ resonant frequency measurement of the microcantilever and micromirror, the Young’s modulus and shear modulus of the G-Cu composite were 123 and 51 GPa, respectively, approximately 1.25 times greater and 1.22 times greater, respectively, than that of the pure Cu. The electrical resistivity of the G-Cu composite was 2.11 μΩ-cm, which is similar to that of pure Cu~1.89 μΩ-cm. The CTEs of the G-Cu composite thin film were measured to be 13.2 ppm/K, which is approximately 77% of the CTE of pure Cu due to the negative CTE of the multilayer graphene, and 30% of the thermal mismatch stress can be reduced by applying G-Cu composite instead of Cu. We demonstrated the engineering in situ mechanical strengthening and thermal expansion reduction of the G-Cu composite using the exfoliated graphene and reinforcement of the graphene in Cu. We believe that the G-Cu composite with excellent behaviors presented in this study can be used as an interconnection material instead of Cu for high-density integration applications.

## Materials and methods

### Electrochemical exfoliation of graphene

Graphene fragments were obtained from the electrochemical exfoliation of graphite in a (NH_4_)_2_SO_4_ aqueous solution as an electrolyte^9,26^. The commercial graphite sheet (EYGS091205, Panasonic Co.,) was used as a working electrode, and a Pt wire was used as the counter electrode. The exfoliation was performed by applying 10 V voltage for 5 min on graphite in the 100 mL electrolyte with a concentration of 0.1 M. During the electrochemical reaction, the graphite flakes dissociated and dispersed into the electrolyte solution. The exfoliated graphene flakes were collected by vacuum filtration and rinsed with deionized water to remove any residual salts. The flakes were suspended into a 500 mL (1.0 wt%) aqueous PDDA solution and sonicated for 30 min to introduce positive charges on the graphene surface. The graphene flakes were subsequently filtrated by a polytetrafluoroethylene (PTFE) membrane filter with a pore size of 0.2 μm from the suspension. The exfoliated flakes were ultrasonically dispersed in an N-Methyl-2-pyrrolidone (NMP) solution for 1 h and maintained for 48 h. The upper part of the dispersed solution was filtrated for collecting high-quality graphene and the synthesis of the composite, and a small amount of the filtrated graphene was employed for graphene characterization.

### Electroplating of G-Cu composite

The exfoliated graphene flakes were added to a sulfuric acid Cu electroplating solution (MICROFAB Cu520, Electroplating Engineers of Japan Ltd.) with a concentration of 0.28 g/L. The solution was homogeneously sonicated to disperse graphene for 1 h.

The G-Cu composite thin film was deposited on a Si substrate (n-type, 1–10 Ω-cm) with sputter-deposited Ti-Cu seed layers by dispersion electroplating, as shown in Fig. 1. The Si substrate was used as a cathode electrode, and a Cu plate was used as an anode electrode. The thickness of the Si substrate and the Ti-Cu layers are 300 μm and 30/300 nm, respectively. A direct current (DC) density of 18 mA/cm^2^ was provided to perform the electroplating according to the standard Cu electroplating condition. The parameters of the G-Cu composite electroplating are summarized in Table 1.

**Table 1:** Deposition parameters of the G-Cu composite electroplating

Cu	50 g/l
H_2_SO_4_	25 g/l
Cl^−^	40 mg/l
Graphene	0.28 g/l
Current density	18 mA/cm^2^
Temperature	25 °C

### Fabrication of G-Cu composite microstructures

**Fig. 6:**
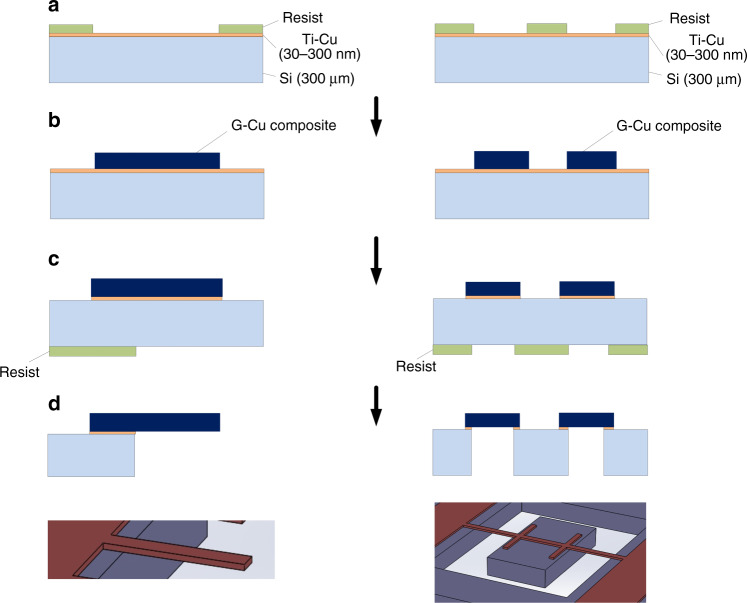
Fabrication process of a G-Cu composite cantilever and micromirror array. **a** Ti-Cu seed layer sputtering on a Si wafer and photolithography for electroplating; **b** G-Cu composite electroplating and resist removal; **c** Surface grinding, Ti-Cu etching and backside photolithography for patterning of the cantilever and micromirror; and **d** DRIE, resist removing and Ti-Cu etching

The G-Cu composite cantilever and micromirror array were constructed using an Si substrate with a thickness of 300 μm. The fabrication process of the cantilever and micromirror is schematically shown in Fig. 6. For reference, a pure Cu cantilever and micromirror array with the same size of the composite cantilever and micromirror array were fabricated by the same processes. The detailed process is described as follows: (a) Titanium (Ti)-Cu (30 nm- and 300 nm-thick) thin films were deposited by sputtering on the Si substrate as adhesion and seed layers. The cantilever and micromirror array patterns were formed on the Si substrate by photolithography. (b) The G-Cu composite with a thickness of 20 μm was deposited on the patterned substrate by electroplating, as described in the electroplating section. The resist was removed. (c) The G-Cu composite surface was grinded using a surface planer (DAS8920, DISCO corporation) with a diamond bit to form a flat surface with a roughness of Ra <1 μm, and the Ti-Cu thin films on the surface were etched by ion beam milling. The back side of the Si substrate was patterned by photolithography. (d) A pattern was etched on the Si substrate underneath the 11.5 μm-thick cantilever and micromirror by deep reactive ion etching (DRIE) to release the microstructures. After removing the resist, the Ti-Cu thin films were removed by wet etching.

### Characterization methods

The nanostructure of the exfoliated graphene was observed using TEM (JEOL 2000F) with 200 kV accelerating voltage. A small amount of the exfoliated and NMP-dispersed graphene was dropped on a copper grid stage and dried before TEM observation. Several graphene sheets with curled edges were chosen to observe the layer stack, and the normal incidence SAED patterns were observed to determine the graphene quality.

The surface morphology of the G-Cu composite was observed by a field emission-scanning electron microscope (FE-SEM, Hitachi SU70). The elemental composition of the G-Cu composite was analyzed by EDS (Oxford Instrument). The electrical resistivity of the G-Cu composite thin films was measured by a four-point probe system after surface grinding. In addition, the CTE of the composite was measured by curvature radius measurement of the composite thin film on the Si substrate using an optical interferometer (Polytech Co., Ltd.). The thermal stress is estimated using Storney’s formula^33^. The composite thin film with a thickness of 7 μm was deposited on a 200 μm-thick Si substrate for the curvature radius measurement.

## Supplementary information

supplemental material
