# Supplementary material for: Mechanically strengthened graphene-Cu composite with reduced thermal expansion towards interconnect applications
Source: Microsyst Nanoeng. 2019 May 20;5:20. doi: 10.1038/s41378-019-0059-0 (PMC6526160; doi:10.1038/s41378-019-0059-0)
Supplement: Supplementary file 1 — supplemental material [file 41378_2019_59_MOESM1_ESM.docx]

**Supporting Information**

Mechanically Strengthened Graphene-Cu Composite with Reduced Thermal Expansion towards Interconnect Applications

Zhonglie An,^1,*^ Jinhua Li,^1^ Akio Kikuchi,^1^ Zhuqing Wang,^2^ Yonggang Jiang,^3^ and Takahito Ono^1,*^

^1^ Graduate School of Engineering, Tohoku University, Aramaki-Aza-Aoba 6-6-01, Aoba-ku, Sendai, 980-8579, Japan

^2^ Research Institute for Engineering and Technology, Tohoku Gakuin University, Tagajo, 985-8537, Japan

^3^ School of Mechanical Engineering and Automation, Beihang University, Beijing, 100191, PR China

*(Z. An) [zhonglie18@gmail.com](mailto:zhonglie@nme.mech.tohoku.ac.jp);

(T. Ono) [ono@nme.mech.tohoku.ac.jp](mailto:ono@nme.mech.tohoku.ac.jp)

**Supplementary Figures**


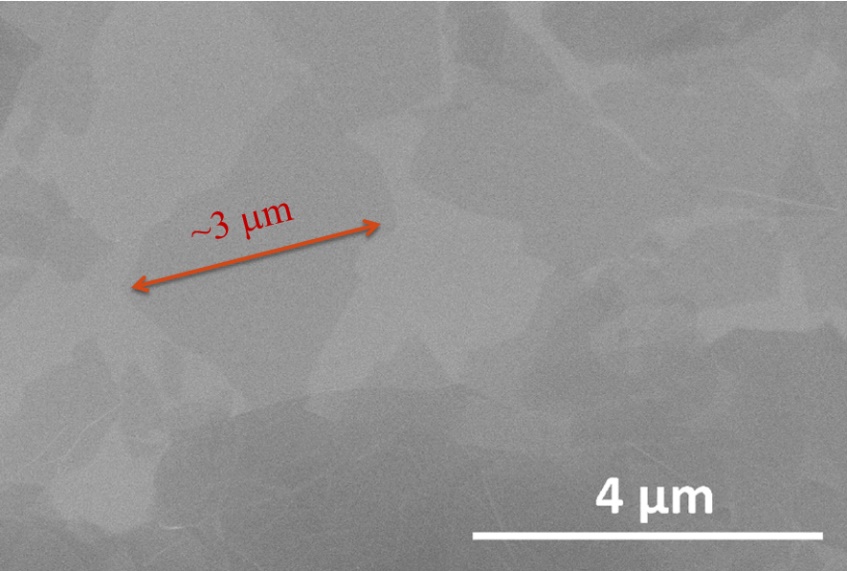


Figure F1. SEM image of the electrochemically exfoliated graphene flakes on Si substrate.


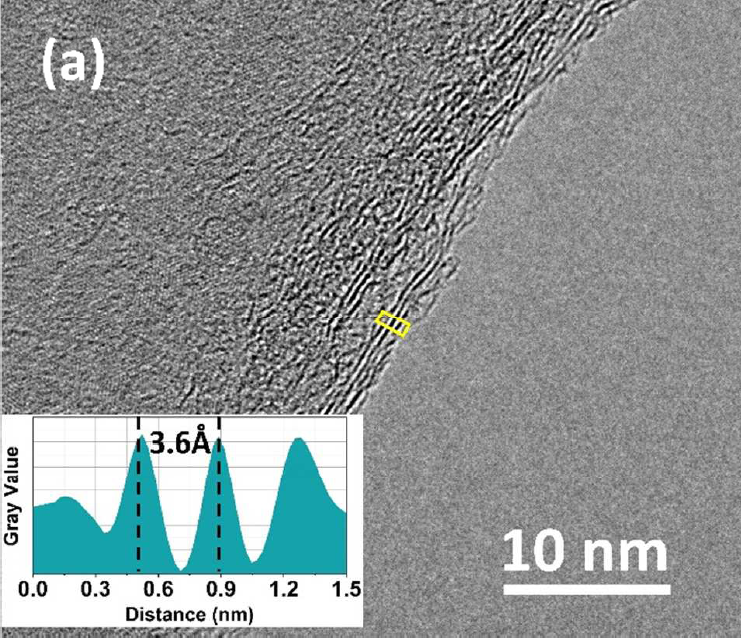


(a)


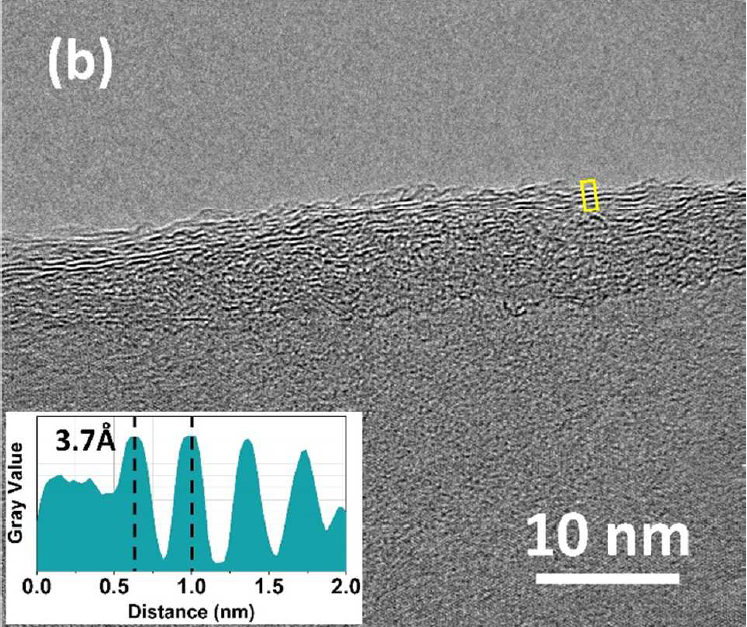


(b)

Figure F2. High magnification TEM images of curled edge in graphene sheets with (a) 3 layers and (b) 4 layers. Insets: line profile based on grey value contrast from the graphene edge in the yellow box with an interlayer distance of ~3.7 Å.


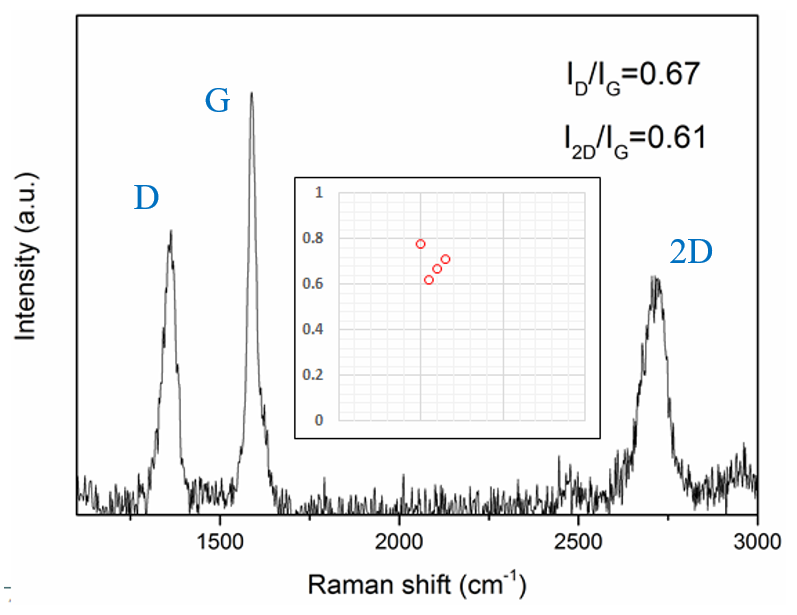


Figure F3. Representative Raman spectra of a graphene flake.

G-Cu composite thin film was electrodeposited on a 200 μm-thick Si substrate and the surface was grinded using surface planer (DAS8920, DISCO corporation) with a diamond bit to form flat surface with a roughness of Ra < 1 μm. The dimensions of the composite thin film and Si substrate were 18 mm×18 mm and 20 mm×20 mm, respectively. The thickness of the thin film was measured by surface profiler as the scanning direction was shown in Fig. F4 (a). One of the experimental results among 6 times of measurement was shown in Fig. F4 (b). The average values for every measurement were 6.9, 7.0, 7.0, 6.8, 7.1, 7.0 μm, totally averaged to be 7.0+0.1/-0.2 μm.


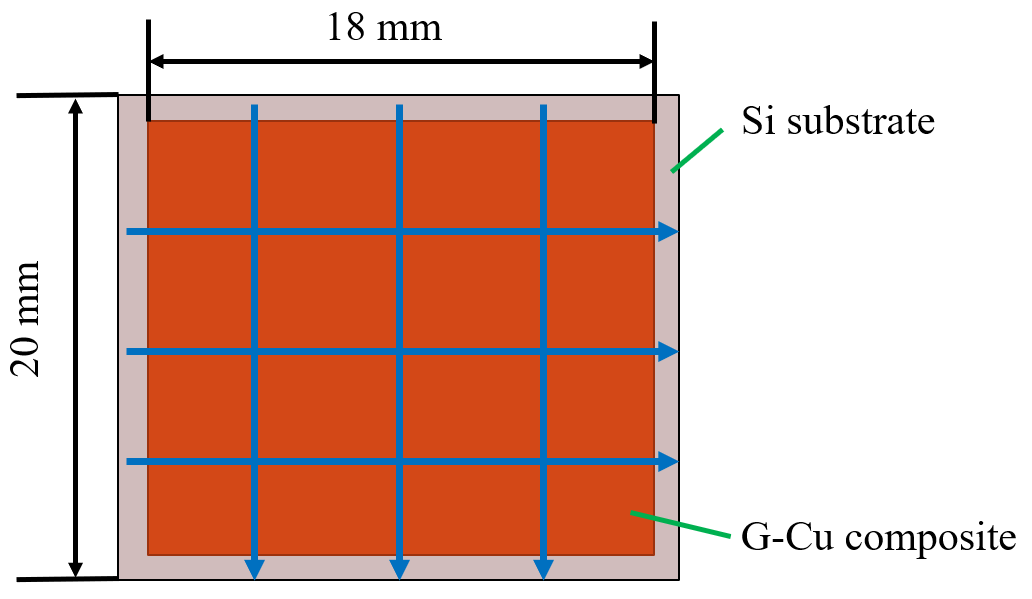


(a)


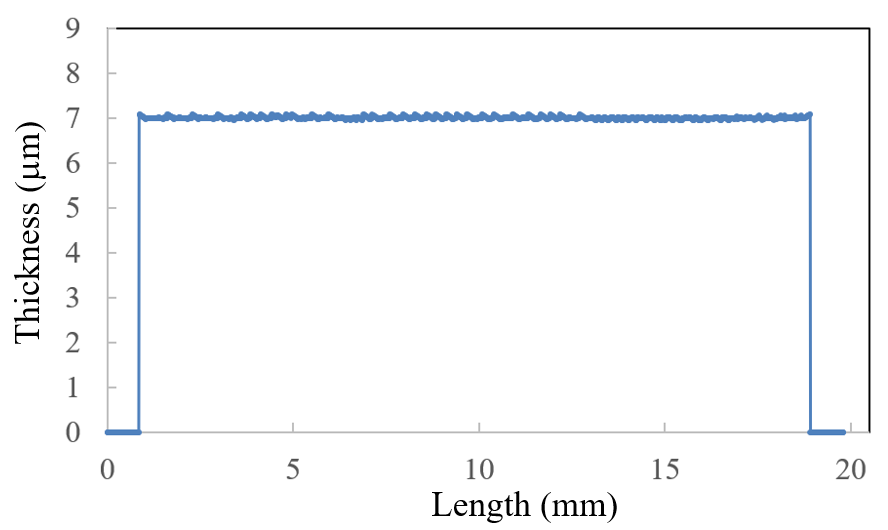


(b)

Figure F4. (a) Schematic of thickness measurement of G-Cu composite thin film on Si substrate and scanning direction, (b) One of the measurement data among 6 times measurement.

**Supplementary Table S1**

**Table S1** –List of cantilever dimension, resonant frequency and Young’s modulus for G-Cu composite and pure Cu cantilevers.


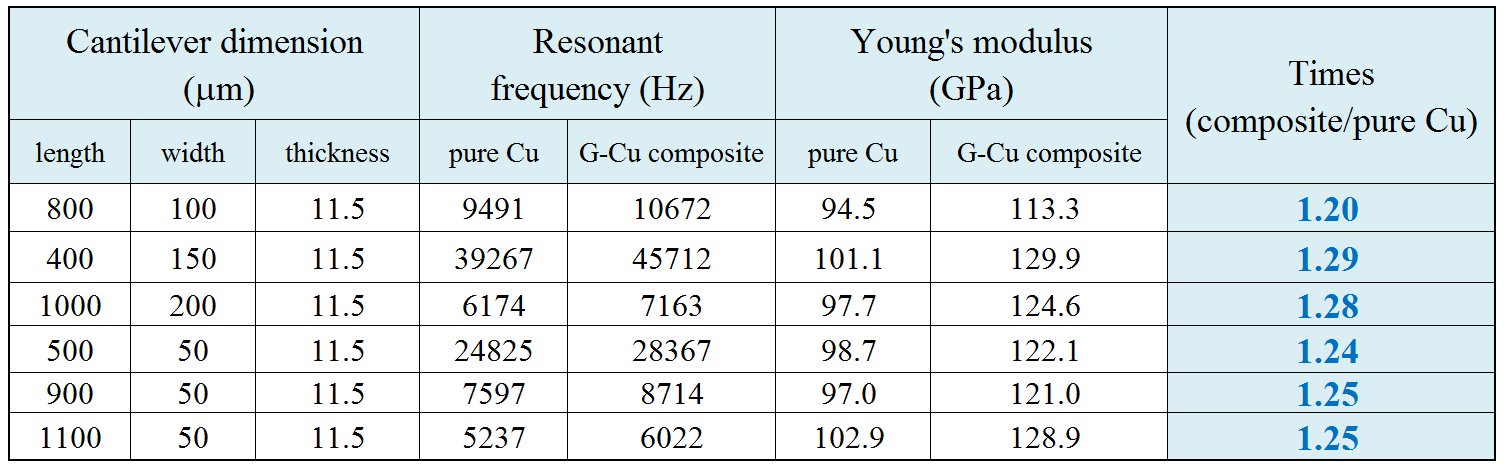


**Supplementary Table S2**

**Table S2** –List of micromirror dimension, torsional beam dimension, resonant frequency of micromirror and shear modulus for G-Cu composite and pure Cu beams.


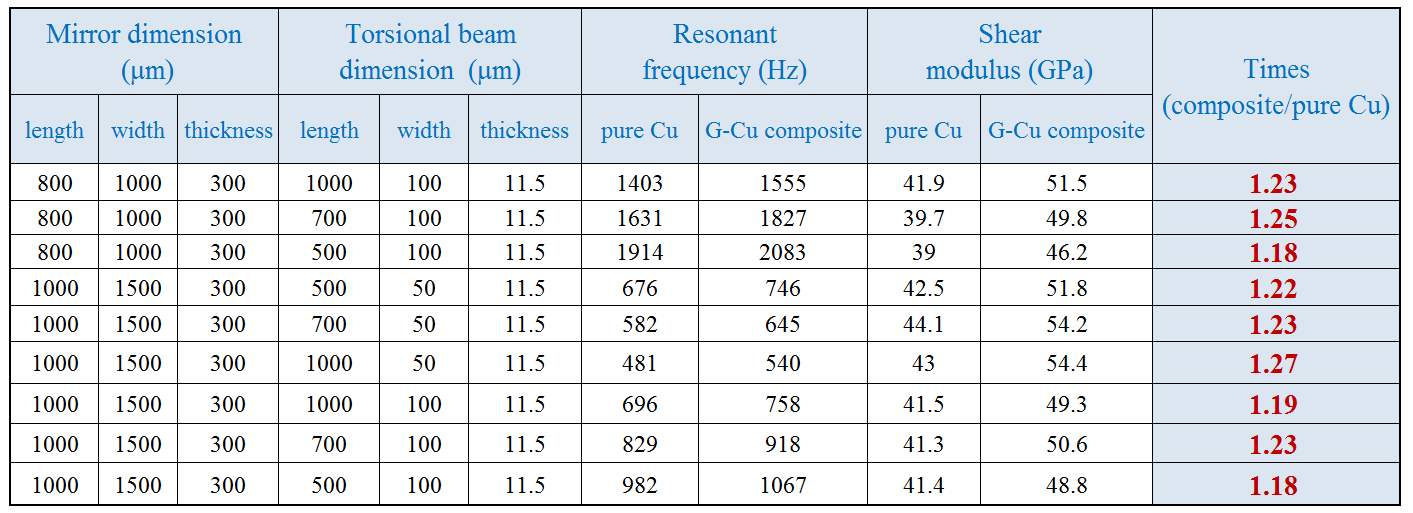


Rotating angles of both Si micromirrors at resonant frequencies are measured in the air atmosphere by measuring the reflected laser line angle on a screen^1^ and summarized in supporting information (Table S3). Rotating angle of the G-Cu composite beam micromirror is smaller than that of the pure Cu beam micromirror in each dimension and average rotating angles for the G-Cu composite beam and pure Cu beam are 14^o^ and 23^o^, respectively. As a brief comparison, it is thought that the G-Cu composite beam is hard to rotate than pure Cu beam because shear modulus and spring constant of the composite beam is increased^1^ due to the embedment of graphene. The smaller rotating angle is also rationalized by the increased mechanical strength of the composite since the addition of graphene would increase the composite tensile strength.

**Supplementary Table S3**

**Table S3** –List of rotating angle of micromirrors with G-Cu composite and pure Cu beams.


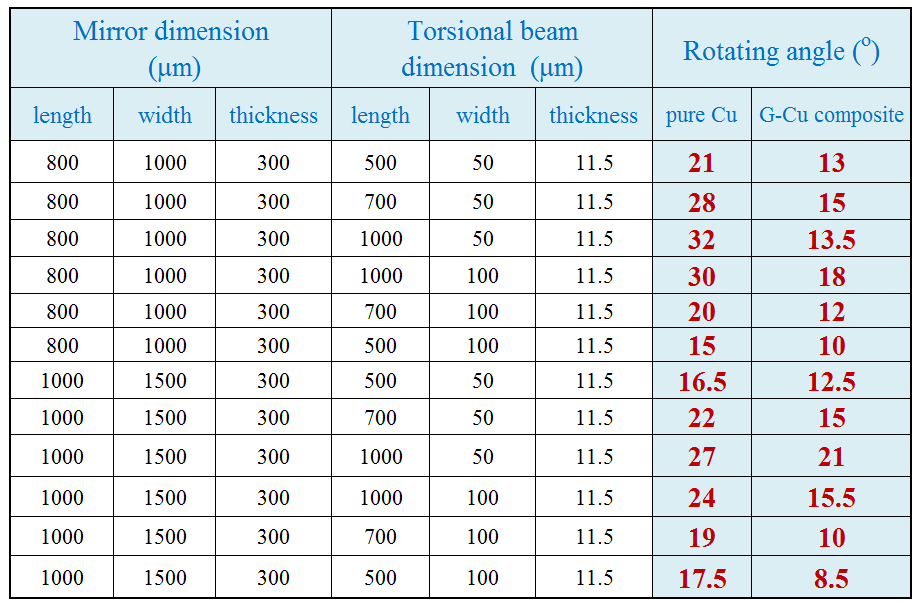


The thermal conductivity of the G-Cu composite thin film was measured at in-plane direction by contactless thermal diffusivity measurement using a thermowave analyzer (TA, Bethel Co., Ltd.). The composite thin film with a thickness of 25 μm was deposited on a Si substrate by electroplating and the Si substrate was etched by RIE to release the thin film. A pure Cu thin film was also prepared with the same method for reference. In this measurement, samples were heated by periodic laser radiation and phase delay of temperature propagation along the in-plane direction with 10 positions were recorded for confirming measurement reliability. The thermal diffusivity *α* is described as

$\alpha=\frac{\pi fl^{2}}{\theta^{2}}$, (1)

where *f* is frequency, *l* is distance between laser radiation point and phase measurement position and *θ* is phase. 6 times measurement for each sample were conducted and average thermal diffusivity values were obtained as summarized in Table S4. The average thermal diffusivity of the G-Cu composite thin film and pure Cu thin film were measured to be 8.3×10^-5^ m^2^/s and 1.1×10^-4^ m^2^/s, respectively. The thermal conductivity *λ* is given by

$\lambda=\alpha\rho c$, (2)

where ρ is density and *c* is specific heat capacity. Since the graphene content in the composite is much less than Cu content, the specific heat capacity of the composite is supposed to be the same with that of the pure Cu based on the theoretical specific heat capacity of composite material.^2^

The average thermal conductivity of the G-Cu composite thin film and pure Cu thin film were calculated to be 265 W/mK and 380 W/mK, respectively. In comparison, the thermal conductivity of the composite is obviously lower than that of the pure Cu. This result indicates that the effect of randomly imbedded graphene in the G-Cu composite did not clearly emerge, resulting in the obviously decreased thermal conductivity from the pure Cu thin film. The conductivity decreasing could be considered by two main reasons. First, graphene thermal coupling to other materials determines thermal boundary resistance at the bonding interface and the thermal boundary resistance at the graphene-Cu interface shows relatively high value.^3, 4^ Therefore, the embedded graphene reduced heat transfer inside the composite and between Cu matrix, resulting in the low thermal conductivity. Second, graphene surface was surrounded with PDDA and bonded with Cu matrix. Therefore, thermal resistance of the composite between graphene and Cu matrix would be increased compared to that of pure Cu and reduced heat transfer inside the composite. Moreover, considering that the thermal conductivity of Cu is naturally very high, the presence of graphene in the Cu matrix significantly affects the heat transfer based on the two-dimensional structure alignment and surface bonding with matrix.

**Supplementary Table S4**

**Table S4** –Lists of thermal diffusivity measured by Thermowave Analyzer and thermal conductivity calculated with density and specific heat for G-Cu composite and pure Cu thin films.

**G-Cu composite:**

| Measurement time | | Thermal diffusivity (m^2^s^-1^) | Density (Kgm^-2^) | Specific heat  (JKg^-1^K^-1^) | Thermal conductivity (Wm^-1^K^-1^) |
| --- | --- | --- | --- | --- | --- |
| 1 | 8.17×10^-5^ | | 8.4 | 380 | 261 |
| 2 | 8.49×10^-5^ | | 8.4 | 380 | 271 |
| 3 | 8.72×10^-5^ | | 8.4 | 380 | 278 |
| 4 | 8.06×10^-5^ | | 8.4 | 380 | 257 |
| 5 | 8.12×10^-5^ | | 8.4 | 380 | 259 |
| 6 | 8.27×10^-5^ | | 8.4 | 380 | 264 |
| Average | 8.31×10^-5^ | |  |  | 265 |

**Cu:**

| Measurement time | | Thermal diffusivity (m^2^s^-1^) | Density (Kgm^-2^) | Specific heat  (JKg^-1^K^-1^) | Thermal conductivity (Wm^-1^K^-1^) |
| --- | --- | --- | --- | --- | --- |
| 1 | 1.15×10^-4^ | | 8.9 | 380 | 388 |
| 2 | 1.16×10^-4^ | | 8.9 | 380 | 393 |
| 3 | 1.13×10^-4^ | | 8.9 | 380 | 381 |
| 4 | 1.14×10^-4^ | | 8.9 | 380 | 387 |
| 5 | 1.09×10^-4^ | | 8.9 | 380 | 370 |
| 6 | 1.07×10^-4^ | | 8.9 | 380 | 363 |
| Average | 1.12×10^-4^ | |  |  | 380 |

References

1. An, Z. et al. Microstructuring of carbon nanotubes-nickel nanocomposite. *Nanotechnology.* **26**, 195601 (2015).
2. Henderson, J. B., Wiebelt, J. A., Tant, M. R., Moore, G. R. A method for the determination of the specific heat and heat of decomposition of composite materials. *Thermochimica Acta.* **57**, 161-171 (1982).
3. Pop, E., Varshney, V., Roy, A. Thermal properties of graphene: fundamental and applications. *MRS Bulletin.* **37**, 1273-1281 (2012).
4. Shahil, K. M. F. & Balandin, A. A. Thermal properties of graphene and multilayer graphene: applications in thermal interface materials. *Solid state Communications.* **152**, 1331-1340 (2012).
